# Expanded Genetic Codes in Next Generation Sequencing Enable Decontamination and Mitochondrial Enrichment

**DOI:** 10.1371/journal.pone.0096492

**Published:** 2014-05-02

**Authors:** Kevin J. McKernan, Jessica Spangler, Lei Zhang, Vasisht Tadigotla, Stephen McLaughlin, Jason Warner, Amir Zare, Richard G. Boles

**Affiliations:** Courtagen Life Sciences, Woburn, Massachusetts, United States of America; Duke University, United States of America

## Abstract

We have developed a PCR method, coined Déjà vu PCR, that utilizes six nucleotides in PCR with two methyl specific restriction enzymes that respectively digest these additional nucleotides. Use of this enzyme-and-nucleotide combination enables what we term a “DNA diode”, where DNA can advance in a laboratory in only one direction and cannot feedback into upstream assays. Here we describe aspects of this method that enable consecutive amplification with the introduction of a 5^th^ and 6^th^ base while simultaneously providing methylation dependent mitochondrial DNA enrichment. These additional nucleotides enable a novel DNA decontamination technique that generates ephemeral and easy to decontaminate DNA.

## Background

Since DNA sequencing data contains both medical information and patient identification data it presents a unique clinical concern for confidentiality. Many next generation sequencing tests are increasingly making use of universal primers that enable amplification of multiple different patients with the same known primer sequences.

A side effect of utilizing universal primers is that subsequent PCR reaction setups are easily contaminated with PCR products from a previous amplification reaction. A second risk in using universal primers is that it hypothetically affords easier theft of patient medical information as the primers required to amplify a patient contaminant from laboratory equipment or trash are well known (i.e. Illumina Primers). To enable easy destruction of clinical DNA, laboratories have traditionally utilized dUTP in PCR to generate PCR products that are different from genomic DNA and are specifically cleavable with uracil DNA glycosylase (UDG) [1]. Using these methods, only the PCR products that contain uracil are enzymatically digested; therefore, any contaminating PCR products can be digested with no risk of destroying the target DNA about to be amplified. Unfortunately, uracilated DNA is not amplified well with widely-used emulsion or cluster PCR kits, due to the use of uracil-illiterate polymerases in most next generation sequencing platforms [2].

To address this deficit, DREAM PCR replaces this uracil base with the 5^th^ base methylcytosine, as most polymerases are methylcytosine-literate and will efficiently incorporate this base into a PCR product [3]. In addition to 5-methylcytosine (5me-dCTP), the recently described “6^th^ base” 5-hydroxymethylcytosine (5hme-dCTP) has been the topic of investigation, and many enzymes exist which differentially digest or capture 5-hydroxymethylcytosine [4], [5]. Due to its unique biochemical properties, techniques that differentially detect 5hmeC from 5meC have been the topic of intense focus [6]–[11], making this an ideal amplification nucleotide to augment DREAM PCR. Both of these methylated nucleotides exist at different frequencies in human genomic DNA [12] and can influence DREAM PCR assay design.

To enable selective serial digestion of the two nucleotides, DREAM PCR substitutes the methyl-specific endonucleases MspJI and AbaSI in place of UDG. MspJI digests heavily methylated PCR products differentially than lightly methylated substrate genomic DNA, and thus it has a preference for digesting double stranded methylated DNA over single stranded lightly methylated circular gDNA presented with a Haloplex exome capture system(Agilent) [3]. This is an important distinction considering the hypermethylated nature of natural CpG islands. Assays targeting CpG islands for sequencing without the single stranded circularization techniques deployed in a Haloplex reaction may choose to use 5-hydroxymethylcytosine as the first amplification nucleotide since its native frequency in gDNA is far lower than the native 5-methylcytosine and thus would better distinguish a contaminant amplicon from a genomic DNA target. For the application of mtDNA sequencing, genomic methyl depletion is preferred due to its concomitant depletion of methylated Nuclear MiTochondrial sequences or NUMTs.

Incorporation of 5-hydroxymethylcytosine enables serial PCR steps to be performed, each with a different 5^th^ base and each respectively digestable with unique enzymes (5meCTP+ MspJI and 5hme-CTP+AbaSI). Such a method offers unique decontamination solutions for more complex massively parallel DNA sequencing workflows requiring more than one amplification step.

## Results and Discussion

### Consecutive amplification utilizes a 6^th^ base

Several clinically relevant next generation sequencing assays require two serial amplification steps [13], [14]. Techniques designed to identify long range genomic phasing often employ whole genome amplification (WGA) before using a more directed PCR approach [15]. In addition, some exome capture techniques require a pre-capture PCR and a post-capture PCR step [16]–[20]. In applications that require serial PCR, one has to consider which amplification step should include the decontaminating methylated cytosine? We chose to use 16 kb long range PCR (LR-PCR) to amplify the whole mitochondrial genome for subsequent transposon-mediated library construction [21], followed by a secondary 12-cycle amplification step (Nextera PCR reaction) using universal Illumina primers [21].

For serial amplification procedures utilizing universal primers, it would be ideal if two different digestible nucleotides were available for exclusive use in respective amplifications. 5me-dCTP and 5hme-dCTP fit this requirement. Both of these nucleotides are commercially available (Trilink); very recently, the enzyme AbaSI also became available (NEB), and is useful as it selectively digests 5hmeC without digesting 5meC [22]. Both enzymes are heat inactivated and thus remain inactive after the first cycle of PCR.

**Figure 1:**
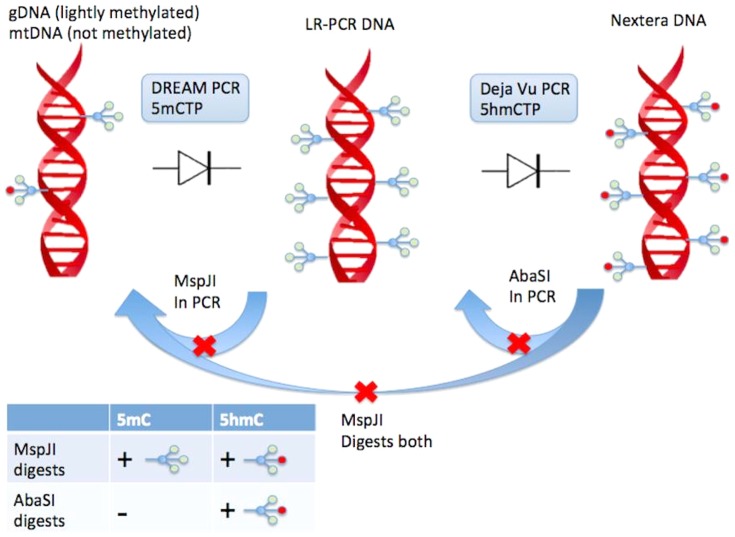
DREAM PCR and Déjà vu PCR makes use of what we have termed a “DNA diode” where enzymes that specifically digest 5^th^ and 6^th^ bases respectively are leveraged to ensure complex serial amplification steps can be performed contamination free without physical isolation of lab equipment. Both enzymes are heat inactivated and do not show activity post PCR. Any hmeC products cannot contaminate the Nextera reaction setup as AbaSI is present to selectively digest hmeC-DNA while leaving the target meC DNA intact. Likewise, any Nextera DNA contaminating the LR-PCR setup will be digested by MspJI since it that targets both forms of methylation.

Decontamination techniques work best when the target to be amplified is different than the product or potential contaminant. If 5me-dCTP exists in the first LR-PCR product, one cannot use MspJI to decontaminate the second Nextera PCR reaction, as MspJI is a methyl-specific restriction enzyme and will digest both the substrate 16 kb target amplicon and any potentially contaminating Nextera PCR products. In order for decontamination to be effective, the post-amplified Nextera contaminants require a nucleotide (here, 5hmeC) that does not exist in the 5meC LR-PCR DNA (Figure 1).

**Figure 2:**
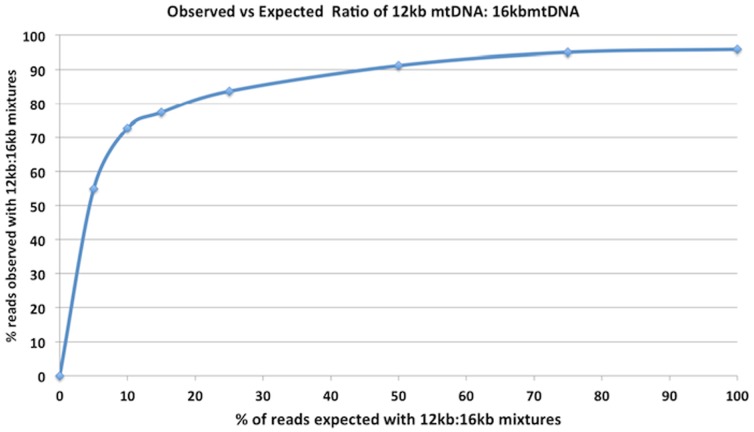
Deleted Mitochondrial DNA hyper-amplifies with LR-PCR. Observed vs Expected coverage of two unique haplogroup mtDNA samples pooled prior to LR-PCR amplification. One 4.5 kb Kearns-Sayre homozygous deleted mtDNA (12.1 kb, KSS mtDNA) sample is mixed with a known wild type mtDNA (16.6 kb, NA12878 mtDNA) sample with a different haplogroup. The KSS mtDNA sample has a unique haplogroup that creates heteroplasmies at expected loci when mixed with a full length mtDNA control. After sequencing the mixtures to 10,000× mean coverage on an Illumina MiSeq V2 system, allele frequencies are measured across a barcoded dilution series where the deleted sample alleles are expected to be seen at 5%,10%,15%,25%,50%,75% of the reads. Plotted is the expected coverage of the KSS mtDNA alleles versus the observed ratio (Y-Axis) of the control mtDNA alleles. This is measured by mapping reads with Bowtie and counting allele frequencies at the haplogroup specific loci. This result is expected in that a multiplexed PCR containing 12.1 kb and16.6 kb molecules will selectively amplify the smaller template. The selective amplification was still observed despite 15 minute extension times applied in PCR. This also highlights the pronounced sensitivity for detecting large deletions in mtDNA samples using LR-PCR.

The described LR-PCR has mitochondria specific primers; thus, contaminants from a Nextera PCR reaction with different universal primers are less likely to create amplifiable contamination. Nevertheless, these Nextera libraries contain mitochondrial DNA inserts, a small portion of which is complementary to the LR-PCR primers. This means secondary amplification artifacts can amplify and impair heteroplasmy detection. In addition to this source of background, deleted mitochondria from other clinical samples can hyper-amplify if co-present with clinical full length mtDNA. Figure 2 demonstrates how a patient with a 4.5 kb mitochondrial deletion known to be associated with Kearns-Sayre syndrome can hyper-amplify (10X) in a foreground of 16.6 Kb target amplification. These two sources of potential contamination underscore the need for decontamination techniques.

### Long range PCR considerations

The use of LR-PCR for massively parallel mitochondrial sequencing has proven to have the most sensitive heteroplasmy and large deletion detection [23]–[25]. This is largely due to LR-PCR's ability to deliver uniform coverage and to limit the amplification of similar NUMT sequences [26] found with methods that use hybridization capture techniques. Nevertheless, LR-PCR methods can be hindered by jumping PCR artifacts with NUMTs, meaning that often the heteroplasmy sensitivity is limited to allele frequencies of 1% or greater, despite the fact that sequencing techniques can deliver accurate allele frequencies far below this [26] with other templates. Since 90% of mtDNA deletions are larger than 2 kb, LR-PCR methods are also prone to hyper-amplification of clinically relevant deleted mtDNA samples [27]–[29].

To address this, we designed a decontamination approach that concurrently depletes methylated NUMTs from the sample. Prior to initiation of PCR, we digest the sample with MspJI as it will digest hyper-methylated dsDNA that can otherwise contaminate the LR-PCR. Exhaustive bisulfite sequencing of mitochondria in several tissues has demonstrated a complete lack of mitochondrial DNA methylation [30], while NUMTs are rapidly methylated in the nuclear genome. This suggests methyl-specific restriction digestion can selectively digest NUMTs and render them non-amplifiable [31], [32]. There are two limitations to this application. First, this methyl depletion step utilized in absence of the selectivity of long range PCR may fail to remove non-methylated NUMTs. Secondly, the minor heteroplasmic non-CpG methylation state of mitochondrial control regions in aged or diseased tissue remains a controversial field [33].

During the first LR-PCR amplification we use a mixture of dCTP and 5-me-dCTP. During the second Nextera PCR we use a mixture of dCTP and 5-hme-dCTP. Since MspJI will digest both 5-meC and 5-hmeC, it will decontaminate the LR-PCR reaction setup of both past LR-PCR product and past Nextera PCR product contaminants while also digesting NUMTs gDNA. It is important to note MspJI's preference of double-stranded DNA over single-stranded DNA and how this preference may alter a given application [34] [35].

**Figure 3:**
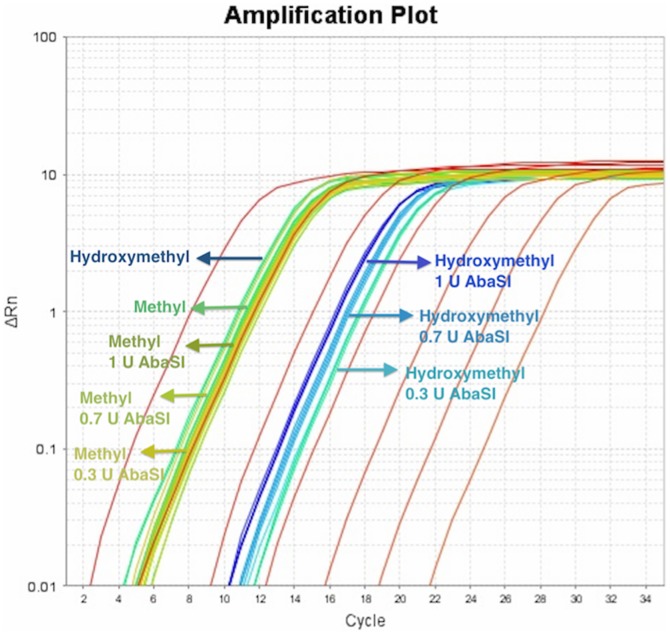
Quantitative PCR of digested and undigested Déjà vu libraries. 120 minute digestion of AbaSI at 25°C on methylated DNA and hydroxymethylated DNA. A 100 fold reduction in background hydroxymethylated DNA is obtained with a 2 hr 25°C digestion with 0.3Units of Enzyme.

After the first LR-PCR and prior to the second Nextera PCR we use AbaSI to digest contaminants as this enzyme only digests 5-hmeC, leaving 5-meC or cytosine intact. In this case, AbaSI will only digest PCR products that contaminate the pre-Nextera sample from the post-secondary PCR process (Figure 3). The second PCR usually contains universal sequencing primers producing small products (700 bp) desired by the limitations of current sequencers. These smaller PCR products can hyper-amplify due to cold PCR or other selective amplification biases and as a result can be over represented. Hyper-amplification of contaminants in PCR a risk in a clinical laboratory testing for heteroplasmy [36].

### Decontamination and optimal sequencing performance

**Figure 4:**
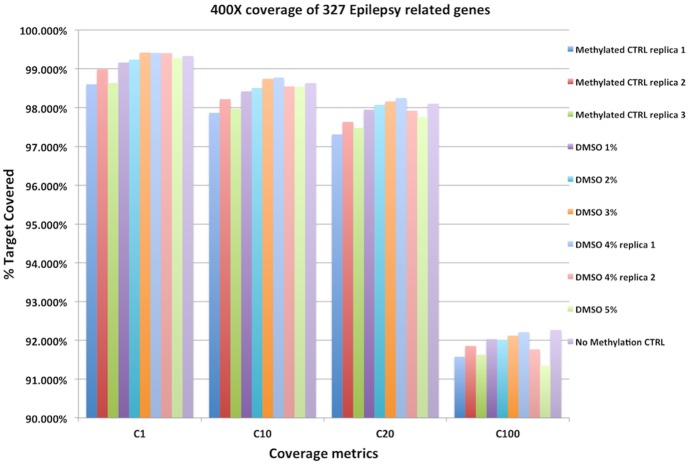
DMSO impact on sequencing methylated libraries. Use of DMSO is estimated to lower the Tm 0.6°C per % according to Von Ashen et al. The use of 4%DMSO improves the C1, C10, C20 and C100 sequencing metrics. All samples were deprecated to 400× coverage to normalize read depth. BEDtools was utilized to calculate C1-C100s coverage statistics. The use of 4% DMSO in PCR with 5mCTP improves the C20 coverage of targets in sequencing panels.

Since 5-meC alters the Tm of DNA by 0.5°C per methylated cytosine, optimizations to the PCR conditions are required [37]. Previous studies with DREAM PCR demonstrated decaying sequencing coverage with increasing concentrations of 5-me-dCTP [3]. Raising the annealing and denaturization temperatures to compensate for 5-meC's impact on Tm exposes DNA to hydrolytic damage [38]. We thus pursued methods that alter the solvation and melting temperature without introducing thermal damage to the DNA. We found that a 3–4% final concentration of DMSO provided optimal sequencing coverage (Figure 4) equal to non-methylated amplification controls.

Of the 354 SNPs identified by GATK (Genome Analysis ToolKit) [39] using the previously published DMSO-free method [3] on NIST (National Institute of Standards and Technology) sample NA12878, 353 variants (99.7% agreement) are found with the 4% DMSO data. The one remaining SNP has evidence of the A>G alternative allele (chr1:116358311) even at a similar allelic ratio (28% vs 31%) but with lower read mapping qualities in the 4% DMSO amplicon. In addition 4% DMSO rescued 7 additional SNPs all present in dbSNP compared to the published methylated SOP. When comparing the 4% DMSO sample to the same control sample run with zero methylation the 4% DMSO provided 358/360 SNPs where the two missing SNPs are C>A and C>T errors (99.4% agreement). This suggests that 4% DMSO in DREAM PCR can compensate for 5meCs known impact on melting temperature.

**Figure 5:**
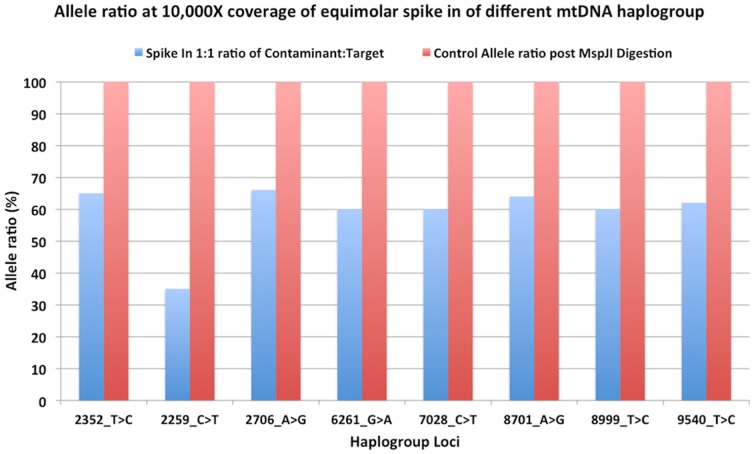
Decontamination effectiveness. To measure decontamination potential we mixed equimolar 5me-dCTP amplified mtDNA into non-methylated Target mtDNA. Methylated and non-methylated DNA were from mtDNA haplogroups differing in 8 loci. Each haplogroup mtDNA sample was barcoded with unique DNA barcodes prior to pooling, decontamination and amplification. Complete decontamination was measured via sequencing the mixed libraries to 10,000× coverage and measuring heteroplasmy levels with and without MspJI decontamination. MspJI digestion removed 100% of expected heteroplasmy contaminants(red) suggesting it can decontaminate up to equimolar contamination events. Undigested pooled libraries were sequenced as a control (blue) and exhibited 35–65% heteroplasmy levels. These artificial heteroplasmies were produced by pooling a methylated mitochondrial Long Range PCR product from a different haplogroup into a non methylated product. This haplogroup is completely removed by the decontamination methods described.

We measured decontamination by spiking in known amounts of DNA contaminant from a different mitochondrial haplogroup. Then, we treated these samples with the respective enzymes and deeply sequenced (10,000×) to measure the percent heteroplasmy of the sample at the haplogroup specific loci. A simple 1 hr digestion was able to remove equimolar contaminating DNA (Figure 5). This assay is limited in that it is only measuring contamination at 8 haplogroup specific loci.

### Mitochondrial enrichment

**Figure 6:**
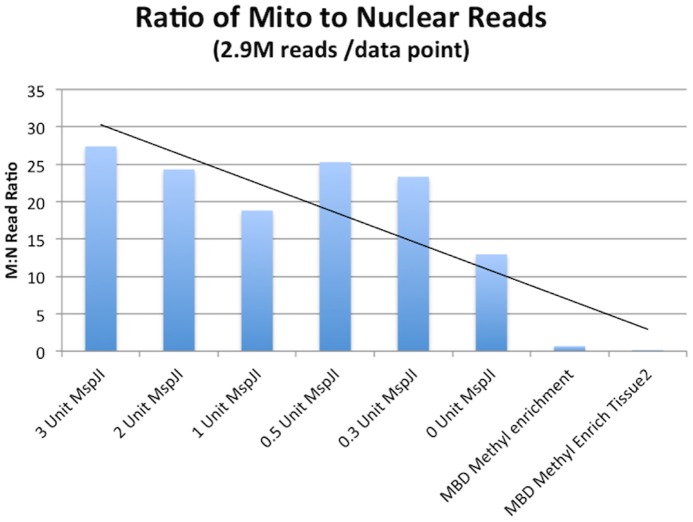
Mitochondrial enrichment. Approximately 2.9 million 250 bp reads were sequenced for each condition. The ratio of Mitochondrial reads to Nuclear reads (M:N ratio) is displayed using Methyl digestion prior to Haloplex capture of targets. X axis displays increasing units of MspJI producing increasing M:N ratios. To confirm the lack of methylation with mtDNA we also performed haloplex capture on a Methyl enrichment fraction derived from Methyl Binding Domain conjugated magnetic particles. (EpiMark, New England Biolabs). Methyl enriched DNA shows a near equimolar 1:1 read ratio despite Control samples showing a 12.3 M:N and MspJI treatment delivering a 25:1 M:N ratio.

**Figure 7:**
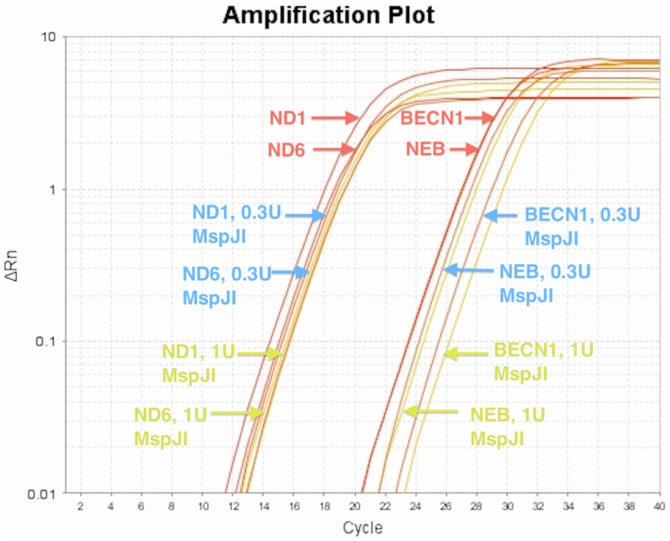
Confirmation of mtDNA copy number with qPCR. SYBR Green Real Time PCR of mtDNA genes ND1 and ND6 estimates mitochondrial copy number at 428 copies next to diploid genes BECN1 and NEB.

To measure the mitochondrial DNA enrichment we designed a Haloplex assay that targeted both the entire mitochondrial genome (320 amplicons) and several nuclear genes in parallel (13,060 amplicons). Genomic DNA was purified and treated with and without MspJI digestion (0,0.3, 0.5, 1,2,3 units of MspJI enzyme). We then sequenced these libraries, and mapped the reads to hg19 to measure the ratio of reads mapping to mitochondrial versus nuclear targets. This mapped read ratio is termed the M:N ratio and is used to estimate enrichment. The M:N ratio in the control sample is 12.3 while the MspJI digested sample has a M:N ratio of 27.3, demonstrating an enrichment of mitochondrial DNA through the digestion of methylated gDNA. We confirmed the M:N ratio of the source DNA with quantitative PCR (Figure 6 & 7).

## Conclusions

Here we report a variation of PCR and sequencing methods incorporating specific enzymatic digestion steps to solve a key reported problem in resolving hydroxymethylcytosine from methylcytosine. Nestor et al. highlighted how challenging this differentiation can be [40] and Wang et al. demonstrated the benefits these enzymes bring to epigenetic studies looking to track the various methylation states with next generation sequencing. Only in recent years has hydroxymethylcytosine been coined the 6^th^ base [8]; this more nuanced view of nucleic acid chemistry raises to question whether the claims of four-nucleotide sequence IDs listed in most gene patents provide sufficient specificity.

Many patents also make claims to any complementary sequence of a defined 4 base sequence ID [41]. Complementarity is defined by Chargaff's rules where the nucleotides base pairing affinity is measured as a function of melting temperature. The use of these expanded nucleotides alters the melting temperature of amplicons significantly in light of Chargaff's rules. Consider a 25mer oligo with the sequence [CATG]_24_ with an adenosine as the 25^th^ 3′ prime base. Changing the 3′ base of this oligo to G,C,T,meC demonstrates a respective shift in Tm of 0.7°C, 0.6°C, 0.2°C, 1.1°C (IDT oligo design tools). This dramatic shift in Tm shown by 5-meC suggests complementarity claims are challenged with the use of 5-me-dCTP in PCR. It is also unclear how Hoogstein base pairing will be interpreted regarding complementarity patent language and if the use of 7-deaza dGTP challenges such claim language since this non-natural nucleotide also alters melting temperature and Hoogstein pairings [41].

Additionally, expanded genetic codes in target amplification can provide both additional error correction opportunities [42], [43] in DNA sequencing and valuable decontamination tools. Since these bases randomly incorporate into GC-rich regions and AbaSI and MspJI cut distal to the methylated base, they can be utilized as a targeting tool for directed fragmentation of recalcitrant GC-rich templates and offer valuable tools for gap closure similar to those methods described by McMurray et al [44].

These results demonstrate additional utility of DREAM PCR in decontaminating more complex amplification procedures than described previously [3]. In addition we underscore the importance of such decontamination techniques for mitochondrial sequencing and the impact of suppressing large deletion hyper-amplification. We also demonstrate a beneficial enrichment of mtDNA by leveraging the lack of methylation in mitochondrial DNA. This addresses a problem with NUMTs contaminating many next-generation mitochondrial sequencing assays previously described and may open the field for accurate sub percentage heteroplasmy sensitivity.

These results likely have relevance for accurate sequencing in any sample that demands low allele frequency quantification like heterogeneous biopsies. Likewise, the results underscore the value in generating ephemeral PCR products. With recent concerns over DNA confidentiality and the ease of de-identification of DNA samples [45], data encryption is becoming a standard in clinical laboratory data management to prevent in-silico contamination or disclosure of DNA sequence [46], [47]. Considering physical DNA can be harvested from 50,000 year old samples [48], a clinical laboratory's trash is a confidentiality exposure point if DNA is not digested or destroyed during testing. Thus methods that eliminate DNA from a clinical laboratory offer attractive and responsible features. In summary, we demonstrate a method that improves DREAM PCR sequencing performance while concurrently providing a more responsible clinical management of patient DNA.

## Materials and Methods

All data for this project has been submitted to the European Nucleotide Archive, http://www.ebi.ac.uk/ena/data/view/PRJEB5732.

### Long-range PCR

PCR setup utilized forward and reverse primers for the ∼16 kb product: mtPCR6F-321-5′TGGCCACAGCACTTAAACACATCTC 3′ and mtPCR6R-16191-5′TGCTGTACTTGCTTGTAAGCATGGG3′. 699 bases are omitted from the D-LOOP due to positive amplification being obtained using those sequences with Rho negative cells (cells with no mitochondria). PCR was performed utilizing 50 ng of gDNA (10 ng/ul). Reaction setup included 1.5 ul of DNA, 5.0 ul of 10 X LA PCR Buffer II, 0.5 ul TaKaRa LA Taq DNA polymerase, 10.65 ul ddH20, and 0.125 ul (50 uM) of each primer with 8.0 ul dNTP mixture (2.5 mM each dNTP where a ratio of 87.5∶12.5 dCTP:5me-dCTP). The 50 ul PCR reaction was cycled with an initial 1 minute denaturization at 94°C and is followed by 30 cycles of 98°C at 10 s, 68°C for 15 minutes. A final 72°C 10 minute extension is performed prior to 4°C hold. PCR products are purified using 75 ul of Ampure (Beckman Genomics).

### Nextera reaction and 5-hydroxymethylcytosine PCR

3 ul (2.5 ng/ul) of the purified LR-PCR product is used in a 10 ul Nextera reaction (1/20^th^X) utilizing 5.0 ul TD, 0.25 ul of TDE, 1.75 ul ddH20 (acronyms according to manufacturers instructions). Samples are incubated for 30 minutes at 55°C followed by a 15 ul Ampure purification. Products are eluted in 25 ul of ddH20 and 10 ul of eluent are used for Nextera PCR with 0.75 ul of each 10 uM primer, 1.25 ul of each Illumina index, 20 ul of 2× Q5 polymerase (New England Biolabs) and 0.75 ul of 5 mM 5-hydroxymethylcytosine (Trilink) with a 4% final DMSO. 12 Cycles of PCR are performed with the following cycling protocol: 72°C for 3 minutes, 98°C for 30 seconds, 12 cycles of 98°C for 10 seconds, 63°C for 30 seconds, 72°C for 1 minute. PCR products are purified using 52.5 ul of Ampure. These products are optionally size selected with a SAGE Sciences Pippin PrepII system in the 600–800 bp size range for 2×250 bp sequencing on a MiSeq V2 sequencer from Illumina according to the manufacturers instructions.

### Decontamination

MspJI digestion is performed with 100 ng DNA, 1 X buffer, 1 X Activator, 1 X BSA, 0.07 U MspJI at 37°C for 30 minutes. The sample is heat killed at 65°C for 20 minutes before initiating PCR.

AbaSI digestion is performed with 1 ng DNA, 1 X buffer, 0.3 U AbaSI, at 25°C for 2 hours. The sample is heat killed at 65°C for 20 minutes before initiating PCR. Figure 3 demonstrates the decontamination with AbaSI with quantitative PCR.

### Enrichment ascertainment

Haloplex assays were designed and amplified according to the manufacturers version 2 instructions (Agilent). MspJI digestion was performed as described above but with various concentrations of enzyme. Experiments were DNA barcoded and sequenced with Illumina MiSeq V2 sequencer with 2×250 bp reads to ensure high mapping quality. All reads were mapped with Bowtie2 and coverage calculations were performed with BEDTools as previously described [3].

The control samples demonstrated a M:N ratio of 12.3. This is very close to theoretical expectations as the size of the amplicon BED file for the mitochondrial and nuclear targets is larger than the desired targets to be sequenced and this presents a M:N amplicon target ratio of 64.8 kb/2.7 Mb or and expected M:N ratio of 0.0236 assuming equimolar copy number. Quantitative PCR suggests a mitochondrial copy number of 428 relative to nuclear control genes. The copy number adjusted M:N is 10 (0.0236*428) and represents the expected M:N ratio we should see in sequencing according to qPCR estimates of the mtDNA in consideration of the in-silico amplicon design. The M:N ratio of the 3 units of MspJI treated gDNA samples is over twice as high (27.3) as the controls (Figure 6). To further confirm these results we used magnetic particles (New England Biolabs, EpiMark) with Methyl Binding Domain (MBD) to methyl capture and sequence a given sample to demonstrate far lower M:N ratios. The MBD particles deliver confirmatory evidence for differential methylation between Mitochondrial and Nuclear DNA (Figure 6).
